# The Enactment of Classroom Justice Through Explicit Instruction: Deciphering the Changes in English as a Foreign Language Teachers’ Perceptions and Practices

**DOI:** 10.3389/fpsyg.2022.821763

**Published:** 2022-02-18

**Authors:** Masoomeh Estaji, Kiyana Zhaleh

**Affiliations:** Department of English Language and Literature, Faculty of Persian Literature and Foreign Languages, Allameh Tabataba’i University, Tehran, Iran

**Keywords:** classroom justice enactment, classroom justice training, English as a foreign language, explicit instruction, just classroom behavior

## Abstract

This mixed methods research study investigated if explicit instruction could affect EFL teachers’ perceptions and practices of classroom justice considering its three-dimensional conceptualization based on the social psychology theories of justice, encompassing the distributive, interactional, and procedural justice. To this end, 77 Iranian English as a Foreign Language (EFL) teachers, chosen through maximum variation sampling, attended a four-session online justice-training course. The data were collected both before and after the course intervention through close- and open-ended questionnaires. Quantitative data analysis results, obtained through running paired samples *t*-tests and Wilcoxon signed ranks tests, indicated that except for the distributive component, the treatment was effective in significantly enhancing the Iranian EFL teachers’ procedural, interactional, and total classroom justice perceptions. Content analysis of the posttest qualitative data, done through MAXQDA, revealed that the participants approved the course usefulness, its significance, and uses of justice enactment strategies in their classroom. Furthermore, they confirmed positive changes in their conceptions and practices of justice because of attending the course and showed enthusiasm in attending more such courses in the future. The convergence of the quantitative and qualitative results in this study demonstrated the effectiveness of the justice-oriented training course for enhancing EFL teachers’ just classroom behaviors. Hence, the results would be fruitful for teacher educators aiming to promote the pre- and in-service EFL teachers’ professional effectiveness through explicit instruction on classroom justice and its use in teacher education programs.

## Introduction

Enacting justice in the domains of classroom learning, interactions, teaching, and assessment is one of the chief responsibilities of the teachers (Chory et al., 2017). This is because the students’ learning attainment, engagement, and their other educational outcomes depend on the degree of perceived fairness in the teachers’ instructional practices and relationships with the students (Kazemi, 2016; Molinari and Mameli, 2017). The extant studies also demonstrate that creating and maintaining a just classroom environment is a primary concern for teachers at various education levels and domains (Horan and Myers, 2009; Berti et al., 2010; Estaji and Zhaleh, 2021a,b). Similarly, the teachers’ just treatment of the students in the instructional context is the learners’ main expectation (Mameli et al., 2018). The critical role that justice plays in the instructors’ successful professional performance is even more visible in the Second/Foreign Language (L2) education, where instruction and learning are essentially relational and social (Pishghadam et al., 2021). In this context, the quality of teacher-student relationships and communication greatly affects the students’ responses and achievements (Mercer and Dörnyei, 2020) since L2 knowledge is learned, co-constructed, and transferred *via* regular interactions and communication of the instructor with L2 learners (Wang et al., 2021).

The emotions, behaviors, and perceptions that the teacher and students hold are intensely intertwined with the learning and teaching processes (Farrell, 2014). Hence, the teachers’ awareness and valuing of the students’ needs and rights — among the most important of which is receiving the teachers’ just treatment (Estaji and Zhaleh, 2021a) — facilitate the L2 learning attainment and success (Mercer and Gkonou, 2020). Against the backdrop of the social psychology theories of justice, it has also been argued that how efficiently teachers incorporate justice when (1) communicating information to and building relationships with the students (i.e., *interactional justice*), (2) enacting classroom procedures and policies (i.e., *procedural justice*), and (3) distributing resources and outcomes among the students (i.e., *distributive justice*; Chory-Assad and Paulsel, 2004; Chory, 2007) can ameliorate or deteriorate the language learning experiences (Estaji and Zhaleh, 2021a,b).

Despite the desideratum for maintaining classroom justice in the spheres of general as well as L2 education, many students around the globe report suffering from their teachers’ unjust behaviour (Čiuladienė and Račelytė, 2016; Chory et al., 2017; Rasooli et al., 2019a). To facilitate the teachers’ just practices, some researchers have advocated that explicit instruction on justice should be the integral aspect of teacher education and preparation programs (Edison, 2015; Sonnleitner and Kovacs, 2020). When the teachers receive explicit instructions, which are systematic, engaging, and direct (Archer and Hughes, 2011) and gain awareness and knowledge regarding the critical tenets of classroom justice, they are more prone to develop their justice perceptions and practices in the classroom.

While the issue of teaching justice to pre- and in-service teachers has not been totally disregarded in educational research — as to date many researchers have focused on instilling the broad concept of social justice into teacher training courses and education programs (e.g., Hytten and Bettez, 2011; Lee, 2011; Dimick, 2012; Kaur, 2012; Edison, 2015; Pantić and Florian, 2015; DeMink-Carthew, 2018) —, except for the experimental studies of Sonnleitner and Kovacs (2020) and Kobs et al. (2021), there is a shortage of empirical studies as for the effect of classroom justice training on the teachers’ professional betterment (Sabbagh and Resh, 2016; Rasooli et al., 2019a). To address this research lacuna, this study sought to determine whether explicit instruction on the central tenets and dimensions of classroom justice, presented in the form of a four-session online training course, could enhance Iranian English as a Foreign Language (EFL) teachers’ perceptions and practices of justice in the instructional context.

### Classroom Justice Conceptualization

At the beginning of the 21st century, classroom justice was introduced for the first time as a concept functioning at both the social and psychological levels, which referred to the degree of perceived fairness in the instructional context (Chory-Assad and Paulsel, 2004). According to the social psychology theories of classroom justice, the concept encompasses three major dimensions since fairness perceptions are formed regarding the extent of justice enacted during the teachers’ allocation of instructional resources and outcomes among students (*distributive justice*), teachers’ deciding on and employment of classroom rules, processes, and procedures (*procedural justice*), and teachers’ interactions, relationships, and information communication with students (*interactional justice*; Resh and Sabbagh, 2016; Chory et al., 2017).

Some scholars conceptualized that for teachers to implement these dimensions in their classes, they are required to follow certain *justice principles* (Cropanzano et al., 2015; Sonnleitner and Kovacs, 2020). Thus, the interactional justice dimension will be actualized when the teachers observe the justice principles of *timeliness* (a well-timed transference of instructional information to the students), *justification/sufficiency* (providing justifiable and sufficient accounts for the classroom rules, behaviors, and decisions), *caring* (attending to the students’ needs, concerns, feelings, and rights), *truthfulness* (making efforts to be considered a reliable, honest, and truthful teacher by the students), *propriety* (acting with decorum toward students), and *respect* (respecting the students’ face, identity, and being as a whole; Rasooli et al., 2019a).

In the same vein, teachers implement the procedural justice dimension by observing the justice principles of *ethicality* (behaving and enacting rules by conforming to prevalent ethical and moral standards), *transparency* (performing class rules and policies in a transparent way), *consistency* (enacting the same class rules and procedures for all the students across time), *bias suppression* (subduing one’s feelings, emotions, and biases when behaving toward the students, evaluating, and making decisions about them), *correctability* (setting class policies and rules which are correctible and modifiable), *accuracy* (setting rules and procedures after obtaining accurate and ample information about the students), *voice* (involving students in making decisions about the class rules and policies), and *reasonableness* (setting reasonable and logical rules and policies; Kazemi and Törnblom, 2008; Cropanzano et al., 2015; Estaji and Zhaleh, 2021a). Finally, the distributive justice dimension happens when teachers follow the *equity* (allotting class resources in accordance to the students’ contributions and performance), *need* (allocating the class resources considering the students’ idiosyncrasies and needs), and *equality* principles of justice (assigning the same amount and kind of resources to all; Jasso et al., 2016; Sabbagh and Schmitt, 2016).

A growing number of researchers have developed the idea and empirically approved that the classroom justice dimensions and principles can be enacted at all classroom domains of *assessment*, *learning*, *teaching*, and *interactions* (Chory et al., 2017; Rasooli et al., 2019a; Estaji and Zhaleh, 2021b). Accordingly, for the first time, in the language education domain, Estaji and Zhaleh (2021a) introduced the three-level conceptualization of classroom justice (i.e., dimensions, principle, and domains). For instance, in the domain of *assessment*, teachers can enact the *international* justice dimension by communicating respectfully (the *respect* principle) with the students during a test, employ the *procedural* justice dimension by applying test procedures equally for all (the *consistency* principle), and realizing the *distributive* justice dimension by distributing test scores that truly represent the students’ efforts and performance (the *equity* principle; Sonnleitner and Kovacs, 2020; Wallace and Qin, 2021).

Similarly, regarding the *learning* domain of classroom, teachers can give equal learning opportunities to all (the *distributive* dimension, *equality* principle), paying attention to the students’ learning difficulties and needs (the *interactional* dimension, *caring* principle), and providing the same instructional materials to all to enhance the students’ learning rates (the *procedural* dimension, *consistency* principle, Rasooli et al., 2019b). As to the *interactional* domain of classroom, teachers can promote justice in teacher-student interactions and relationships by maintaining eye contact with all the students (the *distributive* dimension, *equality* principle), showing care toward learners, listening to them (the *interactional* justice, *caring* principle), and apologizing if behaving inappropriately toward a student (the *procedural* dimension, *correctability* pricniple; Gasser et al., 2018; Rasooli et al., 2019a). Finally, in the *teaching* domain, teachers can incorporate justice in their teaching by allotting differentiated instruction to the students based on their needs (the *distributive* dimension, *need* principle), making instruction clear by designing an accurate syllabus (the *procedural* dimension, *accuracy* principle), and sufficiently explaining the reasons for changing one’s teaching approach (the *interactional* dimension, *sufficiency/justification* principle; Pnevmatikos and Trikkaliotis, 2012; Resh and Sabbagh, 2016). Overall, as the social psychology conceptualization of classroom justice reveals, the concept, being a core value to teachers’ professional effectiveness, penetrates and affects the quality of all spheres of education (Estaji and Zhaleh, 2021b).

### Justice-Oriented Teacher Training Program

As the teacher’s commitment to promoting justice is a desideratum for erecting the cornerstones of a just education system (Mameli et al., 2018; Sabbagh, 2021), some scholars have recommended integrating justice-oriented training into pre- and in-service teacher education/preparation programs (Dimick, 2012; Sonnleitner and Kovacs, 2020). It is expected that training teachers for the rudimental elements of a concept such as classroom justice fosters their justice awareness. “Since teachers become more conscious of the behaviors that the rubric considers desirable and effective, improved practice is often an attractive byproduct of this training” (McClellan et al., 2012, p. 7). To empirically test this argument, previous researchers have attempted to outline the effects of such training on the teachers’ justice perceptions and practices (e.g., Hytten and Bettez, 2011; Lee, 2011; Kaur, 2012; Pantić and Florian, 2015; DeMink-Carthew, 2018). However, these studies focused on the broad concept of social justice rather than classroom justice.

For instance, Edison’s (2015) study outlined a semester-long endeavor to increase global mindedness and social justice awareness in a group of pre-service teachers. As a result, the participants learned how to create lesson plans and units of study around the issues of global citizenship. More theoretically pertained to the concern of the present research are the studies of Kobs et al. (2021) and Sonnleitner and Kovacs (2020). In their experimental study, Kobs et al. (2021) drew the focus of 275 teacher students to the students’ special education needs (the *need* principle) and asked them to evaluate the perceived fairness of various classroom situations presented in the text vignettes. The results indicated that the teacher students’ focus on the students’ behavioral problems, learning difficulties, and the theoretical accounts of interactional and distributive justice underlying the vignettes significantly influenced their justice ratings of student-teacher interactions.

In the same vein, Sonnleitner and Kovacs (2020) explored if administering a Fairness Barometer, which helped identify certain shortcomings and strengths in the instructors’ assessment methods, could improve the teachers’ fairness practices in the assessment domain. To this end, nine teachers were asked to judge their assessment practices considering the different facets of informational and procedural justice. The analysis of the ratings revealed that they could distinguish the various facets of assessment fairness and that certain teacher assessment-related behaviors, such as clarifying the oral exams grading criteria, required improvement. Based on the findings, the authors concluded that the Fairness Barometer could be employed for enhancing the teacher’s assessment fairness literacy in teacher self-development and training.

While the results of such studies are noteworthy, what is still lacking is a comprehensive interventional study promoting the pre- and in-service teachers’ awareness of all the crucial *domains*, *principles*, and *dimensions* of classroom justice enactment conceptualized within the social psychology theories of justice. To narrow this gap, the present study explored if explicit instruction on the essential elements of teacher classroom justice, presented in the form of a four-session online teacher-training course, could enhance Iranian EFL teachers’ perceptions and practices of justice in the L2 instructional context. During explicit instruction, the teacher directs the class, lessons are previously planned and sequenced, explanations and instructions are clear, content is introduced in a step-wise fashion, practice is necessary after each step, there is a high degree of interaction between the teacher and students, and the teacher provides students with systematic feedback and monitoring (Archer and Hughes, 2011).

Since the length of this instructional intervention was constrained, for it to be effective, the course was goal-oriented, planned, direct, teacher-led, and success-oriented. In this study, a convergent parallel mixed methods research design was employed since equal weight was given to both the qualitative and quantitative datasets elicited from the participants before and after the instructions (Creswell, 2008). More particularly, this study aimed to answer two research questions.

To what extent does explicit instruction on classroom justice affect Iranian EFL teachers’ perceptions of classroom justice?

How do Iranian EFL teachers evaluate the usefulness of explicit instruction that they received on classroom justice for their classroom justice perceptions and practices?

## Methodology

### Participants

The participants of the study were 77 Iranian EFL teachers, working part- or full-time at different private language institutes in Iran. The participants were selected through maximum variation sampling (Miles et al., 2014) to, first, reach significant shared patterns emerging from a heterogamous sample, second, to potentially enhance the likelihood of extrapolating the research findings to the population of Iranian EFL teachers (Patton, 2015), and third, to ensure the transferability principle in qualitative research (Nassaji, 2020). To this end, they were chosen from different genders, age levels, teaching experiences, academic levels, English-related majors, cities and provinces of Iran, and teaching levels. The full demographic information of the participants is reported in Table 1.

**Table 1:** The teachers’ demographic information.

Demographic information	Participants (f)	Demographic information	Participants (f)
**Gender**		**Majors**	
*Female*	60	*TEFL*	59
*Male*	17	*English Language and Literature*	13
**Age**		*Applied Linguistics*	3
*Less than 20*	5	*TESOL*	1
*20–29*	52	*Linguistics*	1
*30–39*	14	**Teaching proficiency levels**	
*40–49*	4	*Beginner*	47
*50 or more*	2	*Early intermediate*	52
**Last academic degree**		*Intermediate*	36
*Diploma*	25	*Advanced*	23
*BA*	28	*Proficient*	5
*MA*	20	**Teaching age level**	
*PhD*	4	*Children*	46
**Years of teaching experience**		*Teenagers*	55
*0–4*	48	*Adults*	31
*5–9*	11		
*10–14*	11		
*15–19*	5		
*20–24*	1		
*25 or more*	1		

### Instruments

#### The Demographic Information Scale

A demographic information scale was developed by the present study researchers to collect such non-sensitive information from the participants as their gender, age, academic level, teaching experience, major, teaching level, city, and province.

#### The Needs Analysis Open-Ended Questionnaire

The pre-intervention open-ended questionnaire was developed to do a needs analysis of what the participants perceive of and expect to learn from a justice-oriented teacher training course (Appendix A). The participants’ responses to this questionnaire guided the development of the syllabus, materials, and course instructions.

#### The TCJS

This scale was developed and validated by Zhaleh (2022) in the Iranian EFL education context to measure EFL teachers’ perceptions of their classroom justice practices. The exploratory and confirmatory factor analysis results demonstrated an 18-item scale, measuring the three dimensions of procedural (Items 1, 12, 13, 14, 15, 17, 18), distributive (Items 4, 7, 10, 16), and interactional (Items 2, 3, 5, 6, 8, 9, 11) justice on a five-point Likert scale, ranging from 1 “never” to 5 “always.” In the present study, the participants filled out the scale once before and once after the intervention for within-group comparisons. In the present study, at the pretest phase, Cronbach’s alpha reliability coefficients of 0.71, 0.81, 0.78, and 0.87 were, respectively, reported for the distributive, procedural, interactional, and total classroom justice. Similarly, at the posttest phase of the study, Cronbach’s alpha reliability coefficients of 0.78, 0.83, 0.79, and 0.88 were, respectively, reported for the distributive, procedural, interactional, and total classroom justice.

#### The Follow-Up Open-Ended Questionnaire

The post-intervention follow-up open-ended questionnaire aimed to elicit the teachers’ evaluation regarding the usefulness of the training course that they received for their classroom justice perceptions and practices (Appendix B). There was no restriction on the length of the responses to the questionnaire items. The questionnaire items were checked for their clarity and relevance by four applied linguistics university professors, who were experts in qualitative research studies. After making some changes to the items during several rounds of discussion with the experts, the present study researchers finalized the items and, in this way, ensured the trustworthiness principle of qualitative research and content validity of the questionnaire (Nassaji, 2020).

### Course Design and Materials

Based on the findings obtained from the analysis of the pre-intervention Teacher Classroom Justice Scale (TCJS) and needs analysis open-ended questionnaire responses, a syllabus and materials were developed for a four-session teacher classroom-justice training course. The needs analysis results indicated that although the teachers considered attending teacher-classroom justice workshops and training courses essential for enhancing their professional development and effectiveness, the majority of them had not found an opportunity to participate in such instructional courses and programs. To fulfil the participants’ needs, a course syllabus was designed, based on which the fundamental elements of classroom justice were specified to be instructed to the participants during the subsequent teacher classroom justice-training course (Appendix C). The course materials were prepared in PowerPoint files, entailing different instructional materials, research article findings, frameworks, worksheets, activities, and tasks.

### Data Collection Procedure

In line with the ethical standards in researching on human subjects, the participants initially signed a consent letter (BERA, 2011), demonstrating that they voluntarily took part in the study. The researchers informed the participants of the objective of this research, the nature and duration of their participation, their rights as the study participants, and the anonymity and confidentiality of their data. Furthermore, the participants were explicitly told about the cognitive and behavioral goals and the outcomes of the course and what was expected from them. To carry out the study, the participants primarily responded to the demographic information scale, TCJS, and the needs analysis open-ended questionnaire. Next, they received the four-session training course on teacher classroom justice.

Before running the course, the participants’ opinions were asked regarding its timing and duration. Because of the language teachers’ busy schedule and high workload during the working days of the week, the majority of them preferred the course to be held at the weekend, when they could have more free time to attend the sessions. Thus, the course was run in the form of four consecutive training sessions during a Friday on August 2021, through the Skyroom platform, which lasted for a total of 6 h.

During the first session, the participants received explicit instruction through detailed descriptions in the PowerPoint files on the social psychology theories of classroom justice, its well-established conceptualizations, its main dimensions and principles, key scholars, and major studies conducted around the globe. There were discussions on avenues for future research and practices regarding classroom justice for language teachers, researchers, and practitioners. The objective of this session was to enrich the theoretical and conceptual foundation of the teachers’ knowledge about teacher classroom justice.

From the second session onward, however, a more practice-based approach was taken to instruct classroom justice. Particularly, in the second session, the participants were informed of the importance of teacher classroom justice for language teachers’ effective teaching and professional development. They were also instructed regarding the possibility of enacting justice in all domains of classroom assessment, learning, teaching, and interactions. Then the participants shared their prior experiences of enacting classroom justice principles in their classes. Afterward, many examples of teachers’ just practices in the instructional context were explicated. The participants were subsequently divided into small groups to think of and write down different ways to behave more justly toward their students.

During the third session, the participants were asked to first remember some specific situations that were unfair toward their students, and second, to critically reflect on the reasons/causes of their unjust behaviors. After that, several typical instances of teacher unfairness in the classroom were presented, and the participants were supposed to analyze them in detail and engage in group discussions. Next, they were taught about the detrimental effects of teacher injustice on the students’ behavioral, cognitive, and emotional outcomes based on previous research findings. Next, they were asked to think of and write down the major challenges they had experienced when they wanted to act justly in their classes. They shared their challenges with the course instructor and the other participants during group discussions.

Subsequently, based on research evidence, the trainer made them aware of the typical challenges in the process of incorporating justice. They were then explicitly taught about some effective strategies for overcoming the justice enactment challenges. Then, the participants were asked to recommend some conducive strategies that came to their mind. Finally, with the help of the course trainer, the participants got deeply engaged in devising learning opportunities, activities, tasks, interactions, and teaching and assessment procedures which might seem more just.

In the final session, the participants were invited to ask their questions regarding the different aspects of justice enactment in the classroom, raise their concerns and opinions regarding the effectiveness of the presented course, and engage in discussions and debates around critical injustice issues when dealing with the students in the classroom. The session terminated with the closing remarks of the course instructors, thanking the participants for their attendance, and encouraging them to take more serious steps toward their justice enactment betterment in the future.

**Figure 1:**
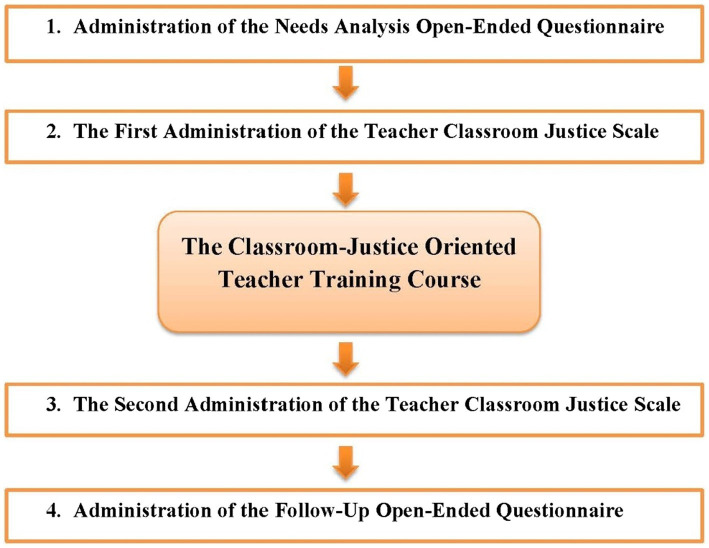
Schematic representation of data collection phases in the present study.

After a week, they responded again to TCJS as well as the follow-up open-ended questionnaire. The phases of data collection are presented in Figure 1.

As all the participating teachers enjoyed sufficient self-perceived language proficiency, the training sessions and data collection processes were all done in English. Both researchers were jointly involved in the data collection, course design, and instruction processes. Since the study was conducted during the COVID-19 pandemic, to prevent spreading the Coronavirus and access the participants more easily, all instructions and data collections were done virtually. The close-ended and open-ended questionnaires were also developed online through Google Forms, and their links were sent to the participants through email, Telegram, or WhatsApp. It took the participants around 10 and 20 min, respectively to respond to the TCJS and open-ended questionnaires at each phase of the study. All the qualitative data collected before and after the course intervention were converted to the Microsoft Word format for content analysis.

### Data Analysis

In line with the recommendation that investigator triangulation — the process of collecting and analyzing the data by more than one person in a study — increases the trustworthiness and credibility of the obtained findings (Patton, 2015); in this research, both researchers were involved in analyzing the data in several joint online sessions. The data analysis for the quantitative phase was done through SPSS (version 24). In this respect, descriptive statistics, normality of the residuals, paired samples *t*-tests, and Wilcoxon signed ranks tests were run on the data.

As for the qualitative data, they were all transferred to MAXQDA (Version 2020), and a mixture of inductive (i.e., obtaining codes, categories, and themes emerging from the data) and deductive creating codes, categories, and themes based on the teacher classroom justice framework proposed by Estaji and Zhaleh (2021a) content analysis approaches (Berg, 2001) were done in an iterative process on the data elicited through the open-ended questionnaires (Miles et al., 2014). According to Estaji and Zhaleh’s (2021a) coding framework, each justice statement can possibly be coded at three levels of justice *dimensions* (i.e., procedural, distributive, or interactional), *principles* (e.g., ethicality, caring, truthfulness, or equity), and *domains* (i.e., teaching, learning, interactions, and assessment). Since there is no clear line between the four domains, the researchers of the present study could not simply categorize many subdomains under just one classroom domain. As an example, the *teacher feedback* subdomain can be categorized under both teaching and learning domains. Accordingly, in the present study, against the notion that the subdomains happen within the teaching, interactions, learning, and assessment domains, the researchers coded the data based on the subdomains.

Following Gao and Zhang’s (2020) qualitative data analysis procedures, the researchers consecutively cleaned up the data, generated open codes, arrived at themes/axial codes, categorized themes, and finally, prepared a detailed report of the analysis. The member checking technique, also known as participant validation, was employed to enhance the credibility of the analysis (Nassaji, 2020). To do so, five participants, who were asked to evaluate the robustness and precision of the generated codes, subthemes, and themes against the actual data, confirmed the accuracy of the analytical process and outputs. To ensure the inter-coder agreement, a university instructor with expertise in qualitative studies in the area of applied linguistics independently coded 20% of the data. Across all the qualitative data, a total of 687 codes were detected. Accordingly, the external coder checked 137 codes. The resulting inter-coder agreement coefficient was 94%, proving the confirmability of the findings (Nassaji, 2020).

## Results

### Comparison of the Teachers’ Classroom Justice Perceptions Before and After the Training Course Through TCJS

To answer the first research question of the study, the participants’ scores obtained from the administration of TCJS as a pretest and posttest were compared. Table 2 shows the descriptive statistics of the obtained results.

**Table 2:** Descriptive statistics of the scores.

		*N*	Minimum	Maximum	Mean	Std. Deviation
Pretest	Distributive	77	11.00	20.00	16.85	2.12
	Procedural	77	18.00	35.00	30.23	4.09
	Interactional	77	21.00	43.00	29.87	3.90
	Total	77	51.00	97.00	76.10	9.59
Posttest	Distributive	77	12.00	23.00	17.07	2.30
	Procedural	77	21.00	35.00	31.54	3.14
	Interactional	77	20.00	35.00	31.35	3.37
	Total	77	53.00	93.00	79.97	7.58

As evident from the Table 2, there were improvements in the scores from pretest to posttest in all parts of the questionnaire. The significance of these improvements had to be checked through running paired samples *t*-tests or the non-parametric equivalent. Before running the tests, the normality of residuals, as the pre-requisite, was checked (Table 3).

**Table 3:** The descriptive statistics of the residuals.

	*N*	Minimum	Maximum	Mean	*SD*	Skewness		
						Statistic	Std. Error	Ratio
Distributive	77	−4.00	7.00	0.22	1.97	0.63	0.27	2.32
Procedural	77	−8.00	9.00	1.31	3.25	0.15	0.27	0.54
Interactional	77	−8.00	10.00	1.48	3.26	−0.08	0.27	−0.32
Total	77	−14.00	36.00	3.87	8.19	1.04	0.27	3.82

As reported in Table 3, the distributive competent had the lowest mean of residual and the interactional one the highest. The overall mean of difference was 3.87, with the standard deviation of 8.19. Moreover, the skewness ratios for the procedural and interactional components of the questionnaire indicated the normality of distribution as the values fell within the legitimate range of ±1.96 (Tabachnick and Fidell, 2013), while the distributive component as well as the total scores were not considered normally distributed. Therefore, the pretest and posttest scores of the datasets with normal distributions of residuals were compared through running paired samples t-test and the ones with non-normal distributions through non-parametric Wilcoxon signed ranks tests. Table 4 shows the results for the paired samples *t*-tests.

**Table 4:** Paired samples *T*-Tests: Comparing procedural and interactional scores from pretest to posttest.

		Paired Differences					T	df	Sig. (2-tailed)
		Mean	*SD*	Std. Error Mean	95% Confidence Interval of the Difference				
					Lower	Upper			
Pair 1	Procedural	1.31	3.25	0.37	0.57	2.05	3.53	76	0.001
Pair 2	Interactional	1.48w	3.26	0.37	0.73	2.22	3.97	76	0.000

Table 4 shows that the difference in both procedural [*t*_(76)_ = 3.35, *p* = 0.001 < 0.05, Cohen’s *d* = 0.348, representing a small effect size] and interactional [*t*_(76)_ = 3.4, *p* = 0.001 < 0.05, Cohen’s *d* = 0.348, representing a small-to-medium effect size] components were significant, indicating that the treatment was effective in improving them. Table 5 shows the results of the non-parametric tests in comparing the distributive and total scores of the participants.

**Table 5:** The Wilcoxon Signed Ranks tests: Comparing distributive and total scores from pretest to posttest.

	Distributive	Total
Z	0.65^a^	3.78^a^
Asymp. Sig. (2-tailed)	0.51	0.00

a Based on positive ranks.

The results reported in Table 5 show that while the treatment failed to make a significant change in the distributive component, the total scores were significantly improved (Z = 3.78, = 0.000 < 0.05, Cohen’s *d* = 0.95, representing a large effect size).

### Exploring the Changes in the Participants’ Perceptions and Practices Through a Follow-Up Open-Ended Questionnaire

To answer the second research question, after attending the course, the participants were asked to complete a follow-up open-ended questionnaire, eliciting their opinions regarding the effectiveness of the course for their classroom justice perceptions and practices. The six themes emerging from the analysis of the data are illustrated in the following sections.

#### The Usefulness of the Teacher Classroom Justice Training Course

The first theme emerging from the qualitative data pertained to Iranian EFL teachers’ opinions regarding the usefulness of the training course that they received on classroom justice. Except for one teacher who did not find the course beneficial and two teachers who had no idea in this regard, 96% of the teachers (*N* = 74) mentioned that the course was beneficial for them. They brought different reasons for the course effectiveness, a few of which are presented in Table 6.

**Table 6:** The teachers’ reasons for the usefulness of the course.

Participant	Excerpt
T9	“*It helped me understand that some of my actions in the class were not right*.”
T14	“*It made me realize that some of my policies were wrong, I have to deal with them differently*.”
T22	“*It made me reflect on my attitude and approach toward language learning and teaching*.”
T30	“*I understood how I should treat all kinds of students to be a just teacher, how to give exams, and how to communicate with students*.”
T36	“*I consider the course one of the best training courses since the concept of justice is really important, but unfortunately long forgotten. Now, I’m familiar with new ideas and strategies for teaching in the classroom. I have better perspectives on the classroom and students. I can enhance my teaching method and help and behave toward students in a better way*.”
T63	“*I realized that I can have a better relationship with my students through using the justice strategies I learned*.”
T76	“*Many ambiguities were resolved in my mind due to the explanations provided in the course*.”

Overall, the teachers’ accounts revealed that they had positive attitudes toward the course and believed that different aspects of the course could enhance their teaching effectiveness.

#### Importance of Classroom Justice to the EFL Teachers

The second theme emerged from the qualitative data when the participants were asked if the idea of being a just teacher became more important to them after attending the course. The results revealed that except for three teachers having no idea and four teachers not considering justice much important, the rest (*N* = 70) considered classroom justice to be more important to them after attending the course. Although many of them mentioned that being a just teacher had been important to them even before attending this course, they explained that the explicit instructions that they received increased the significance of classroom justice for them. They explained that the course brought good understanding about justice into their conscious mind, including “*paying more attention to their behaviors in the class*” (T9), “*preparing an equal opportunity for all”* (T5), “*doing a needs analysis in the first session”* (T6), “*loving one’s class and students more”* (T12), “*considering justice during assessment, learning, teaching, and interactions*” (T33), “*considering respect and impartiality as two of key elements in teaching*” (T56), and “*transferring positive attitude, behaviors, good vibes*” (T75).

#### Changes in the EFL Teachers’ Knowledge of Classroom Justice

**Figure 2:**
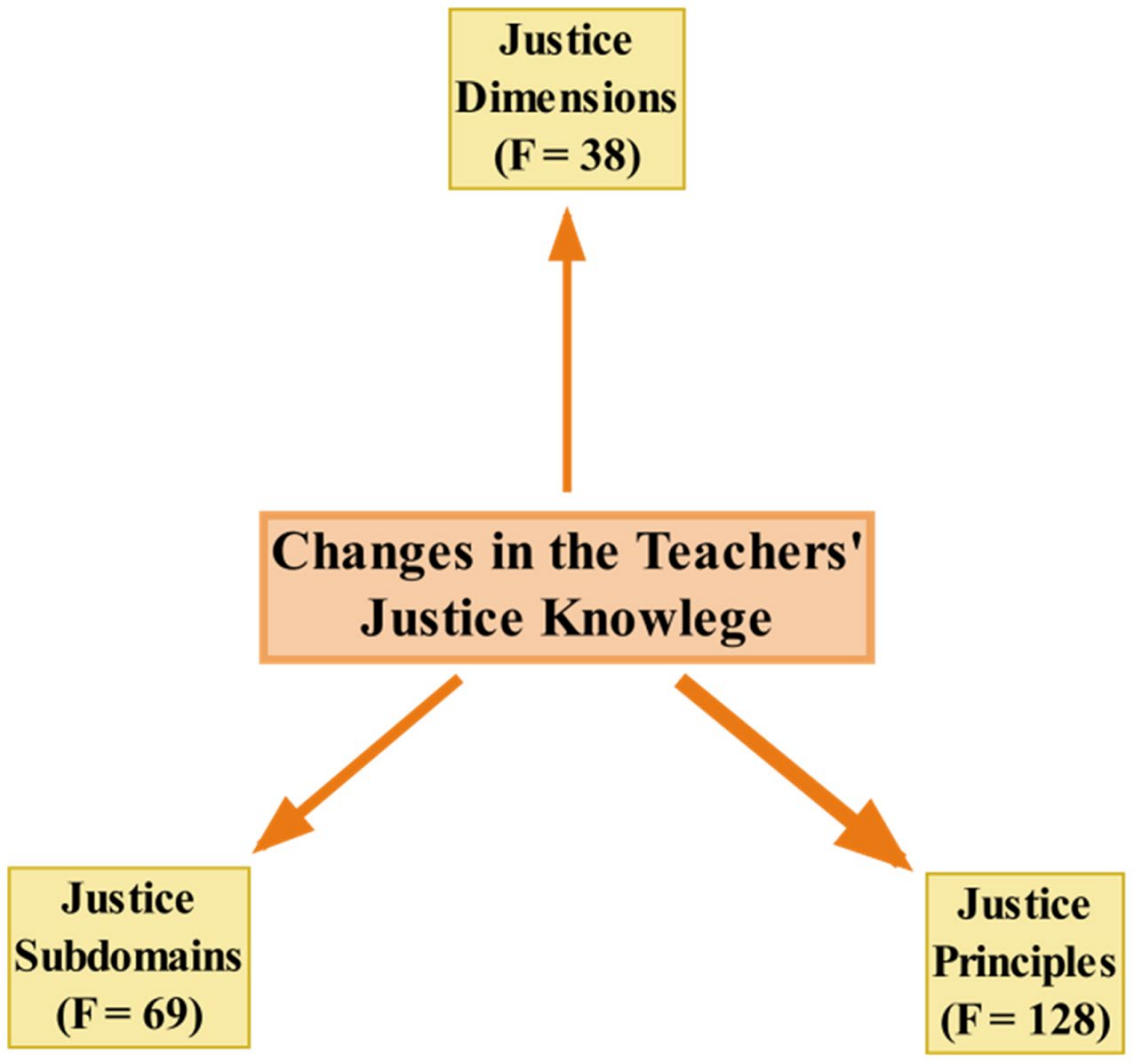
Changes in the teachers’ knowledge of justice.

The third theme emerging from the qualitative data was about the ways that teachers’ knowledge of classroom justice changed because of attending the course. The results indicated that most of the teachers approved the positive changes happening in their understanding and expertise about justice because of attending the course. Figure 2 visually depicts the things that the teachers learned from the course.

As portrayed in Figure 2, the teachers have reported changes in their knowledge of justice principles, subdomains, and dimensions in consequence of attending the course (see Appendix D for the full list of principles, subdomains, and dimensions learned by the teachers with their frequencies). The teachers reported that they learned the most about justice principles (*N* = 118). Their accounts revealed that they gained knowledge about all the principles of equality, need, caring, equity, bias suppression, transparency, respect, consistency, correctability, accuracy, justification, truthfulness, timeliness, propriety, ethicality, voice, and reasonableness. They also reported learning about the various subdomains of justice enactment (*N* = 69), such as class rule and policies, affect and attention, opportunities, assessment and grading, assistance, syllabus design, materials, reward, and feedback. Moreover, their awareness regarding the knowledge of the procedural, interactional, and distributive justice dimensions (*N* = 38) increased.

#### Justice Enactment Strategies Learned From the Training Course

**Figure 3:**
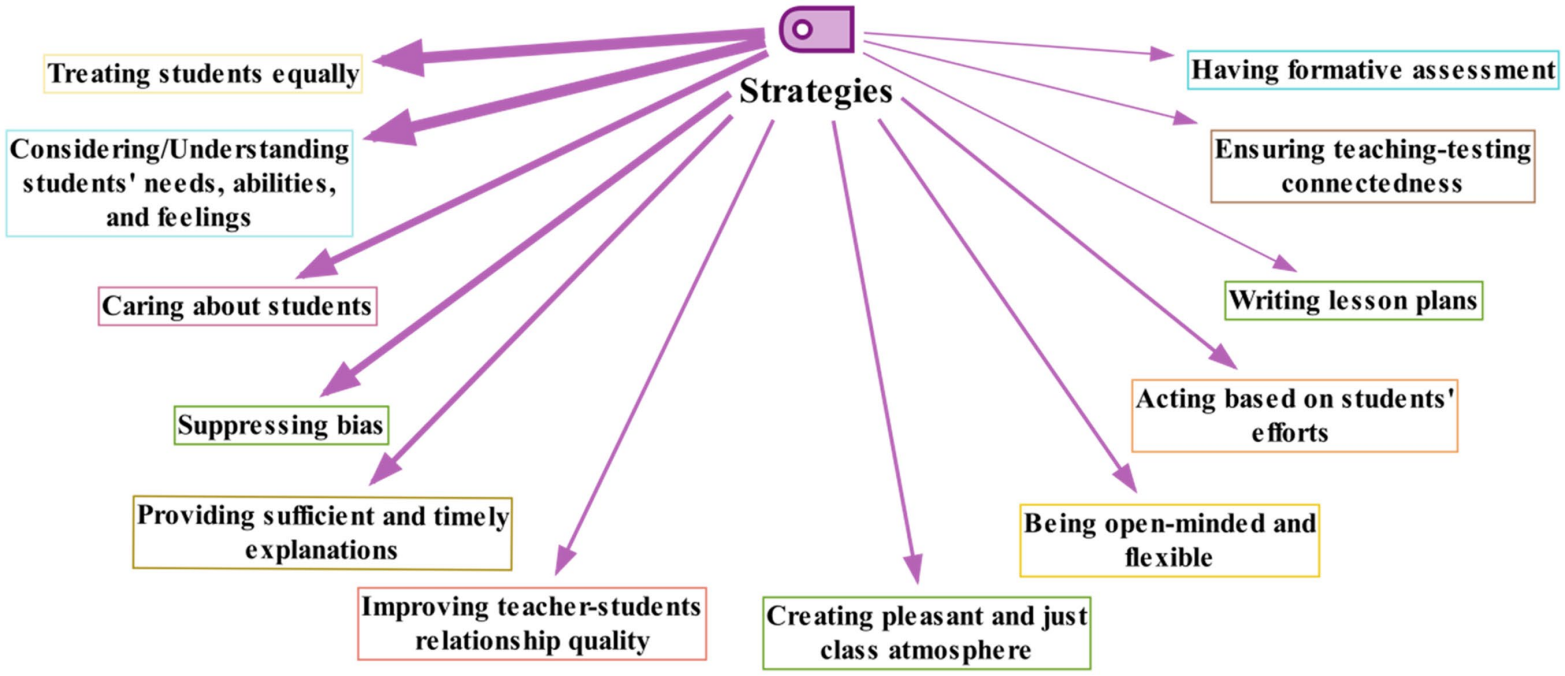
The justice enactment strategies learned by the teachers (Line width indicates frequency).

The teachers reported having learned several useful strategies from the course to act more justly in their classes (Figure 3; Appendix E).

According to Figure 3, among the many strategies mentioned by the teachers, treating students equally (*N* = 28), considering/understanding the students’ needs, abilities, and feelings (*N* = 23), caring about the students (*N* = 14), and suppressing bias (*N* = 13) were the most frequent ones. Some of the less frequently mentioned strategies learned from the course were providing sufficient and timely explanations (*N* = 6), creating a pleasant and just class atmosphere (*N* = 5), improving the quality of teacher-students relationship (*N* = 5), and being open-minded and flexible (*N* = 4).

#### Changes in EFL Teachers’ Practices of Classroom Justice

**Figure 4:**
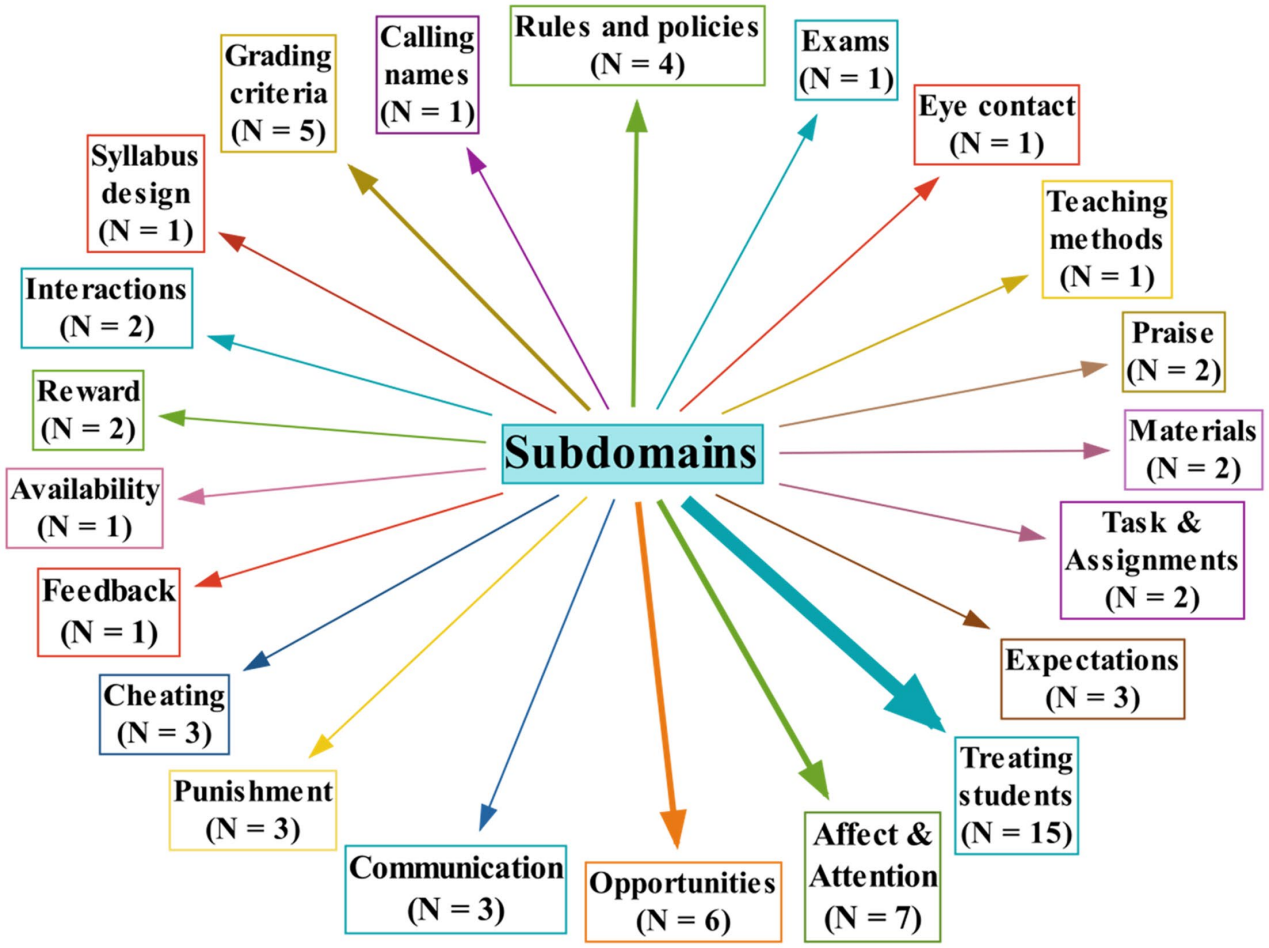
Changes in the teachers’ classroom practices.

Many of the teachers reported that they experienced improvements in different aspects of their classroom behaviors and practices because of attending the course. As presented in Figure 4, the changes happened in many subdomains of the teachers’ classroom practices through enacting the justice principles of caring (*N* = 9), voice (*N* = 2), equality (*N* = 10), equity (*N* = 2), timeliness (*N* = 1), propriety (*N* = 2), bias suppression (*N* = 10), transparency (*N* = 4), justification (*N* = 10), reasonableness (*N* = 2), and correctability (*N* = 1).

Some examples of the positive changes in the teachers’ practices are presented in Table 7.

**Table 7:** Changes in the teachers’ classroom practices.

Participant	Excerpt
T3	“*Before, I was mostly focused on student achievement. Now, I pay more attention to interactions and emotions*.”
T12	“*I try to explain my policies and grading system at the beginning of the semester*.”
T21	“*I understand that if they have a problem and cannot do their homework, I will be more flexible*.”
T27	“*I tried to communicate with students more than before outside of class*.”
T34	“*Now, I ask my students’ opinions anonymously. I give all my students equal time to read texts, speak, and ask questions. I do my best to pay attention to them. I try to understand them and be a good friend to them*.”
T51	“*I tried to accept my students’ ideas about attendance policy*.”
T55	“*I change my behavior when teaching to low-achievers and accept them with all their disabilities and low learning rate*.”
T61	“*Informing students about exams, materials, and my expectations; Before, I did not care much, but now I put more emphasis on these issues*.”
T69	“*I design syllabus according to the capacities of students*.”

#### The EFL Teachers’ Plans for Attending Future Justice Training Courses

Many of the teachers expressed positive attitudes toward attending more teacher-justice training courses and workshops in the future (*N* = 66). However, six teachers found the training sessions comprehensive which sufficiently guided them toward just practices, while the rest (*N* = 5) had no idea about attending future training courses. Overall, these results suggest that Iranian EFL teachers care about their acting justly in the instructional context and deem attending more justice training sessions necessary for meeting their continuing professional development needs.

All in all, the quantitative and qualitative results obtained and analyzed in this study converged and approved that the training course that the Iranian EFL teachers received significantly enhanced their perceptions and practices of classroom justice.

## Discussion

### Discussion of the Main Findings

This mixed methods research study aimed to unravel to what extent and in what ways explicit instruction on teacher classroom dimensions based on the social psychology conceptualization of justice could improve the EFL teachers’ perceptions and practices of justice in language classes. In this respect, 77 Iranian EFL teachers received classroom justice-oriented training during four online sessions, each lasting for one and a half hours. Qualitative and quantitative data were collected from the participants both before and after the intervention. Analysis of the pre-intervention qualitative data, which was a form of needs analysis, informed the design of the syllabus and materials for the course. Within-sample comparison of the pre- and posttest quantitative results indicated that, except for the distributive justice, attending the course significantly improved Iranian EFL teachers’ perceptions of the procedural, interactional, and total classroom justice. This finding is in line with those of Sonnleitner and Kovacs (2020) and Kobs et al. (2021), which showed that increasing the teachers’ awareness of the theoretical underpinnings of distributive, procedural, or interactional justice and their unique principles influence the teachers’ judgments, perceptions, and practices of classroom justice in various subdomains.

Analysis of the post-intervention qualitative data revealed six themes, which converged thoroughly with our quantitative findings. First, the participants mainly considered the classroom justice-training course very fruitful for making them more conscious of their unjust practices, reflecting on their teaching behaviors and practices, and treating the students more justly. This finding complies with the previous researchers’ recommendation for integrating justice-oriented training into pre- and in-service teacher education programs because of their potential usefulness for enhancing the teachers’ professional effectiveness (Dimick, 2012; Edison, 2015; DeMink-Carthew, 2018). It is also in compliance with Crandall and Finn Miller’s (2014) account that teacher training, which involves spending time fortifying one’s teaching skills and sub-skills, receiving peer and instructor support, finding the opportunity to exchange ideas, and engaging in discussions, are helpful as they push teachers toward instructional mastery and amelioration.

Besides, the participants of this study acknowledged that attending the course brought about favorable changes in their knowledge of classroom justice. In this respect, the teachers attained more awareness about the procedural, distributive, and interactional justice dimensions (Chory, 2007; Jasso et al., 2016) and all their principles including equality, need, caring, equity, bias suppression, transparency, respect, consistency, correctability, accuracy, justification, truthfulness, timeliness, propriety, ethicality, voice, and reasonableness (Rasooli et al., 2019a). Similarly, they reported learning about the various subdomains of justice enactment such as class rules and policies, affect and attention, grading criteria, and opportunities.

This finding is quite in congruence with the propositions of social psychology theorists, who conceptualize justice as a multi-dimensional phenomenon (Resh and Sabbagh, 2016; Chory et al., 2017), implemented through unique justice principles (Cropanzano et al., 2015; Rasooli et al., 2019a) in the interactions, learning, assessment, and teaching classroom domains (Horan and Myers, 2009; Estaji and Zhaleh, 2021a). Thus, explicit instruction based on the social psychology conceptualization of classroom justice would be effective for successfully conveying the theoretical knowledge base of classroom justice to EFL teachers (Sonnleitner and Kovacs, 2020).

Further qualitative results uncovered that after the training, the teachers allotted more significance to the issue of enacting justice in language classrooms. It seems that the increase in the teachers’ awareness and knowledge of justice promoted them to consider it a critical classroom aspect, which is in line with the findings of Kobs et al.’s (2021) study. The qualitative data supported this notion when the teachers mentioned that attending the course aided them to better understand the importance of paying attention to one’s unjust behaviors in the class, preparing an equal opportunity for all students, and inculcating justice during one’s assessment, learning, teaching, and interactional practices. This finding approves Derakhshan et al.’s (2020) account that informing teachers of new theories and research evidence during teacher training courses open their eyes to the important aspects of teaching, which were simply overlooked or unattended to before.

In the same vein, the participants reported that besides gaining knowledge of the theoretical aspects of classroom justice, they learned useful and practical justice enactment strategies during the course, such as strategies for treating students equally, considering the students’ needs and feelings, caring about the students, and suppressing one’s biases in the class (Gasser et al., 2018; Rasooli et al., 2019a). Having sufficient knowledge and practical skills regarding a particular area are the pre-requisites to better implementation of justice (Estaji and Zhaleh, 2021b). Thus, when teachers are equipped with a rich instructional repertoire, they are more inclined to practice what they theorize (Derakhshan et al., 2020) and make more informed and opportune decisions in the classroom (Coombe, 2020).

A course like the one proposed in this study helps to narrow the theory-practice gap in the teachers and empowers them to adroitly handle daily justice enactment challenges and hardships (Estaji and Zhaleh, 2021b). Further qualitative findings of the study approved this notion by revealing that the teachers experienced improvements in their actual classroom justice practices after the training by taking such actions as giving all students equal time to read texts, accepting the students’ ideas about attendance policy, and informing them about exams, materials, and ones’ expectations. When teaches are professionally developed by attending training courses, conferences, and workshops, enhanced practice is obviously around the corner (Rimmer and Floyd, 2020).

This more just practice of EFL teachers after the intervention also supported Borg’s (2018) assertion that when the teachers’ continuing professional development needs are met in teacher training courses and workshops, they sense more satisfaction, and thereof, their professional performance is boosted. The enhanced justice practice of EFL teachers found in this study was to some extent expected because, as mentioned by Coombe and Hiasat (2020), gaining literacy in a specific teaching domain promotes the teachers’ taking of appropriate actions to its subsequent enactment in the instructional setting. Finally, the results uncovered that EFL teachers were enthusiastic about attending more such training courses in the future to stay current with new updates and issues about classroom justice. This positive attitude of teachers about continuing their classroom justice learning is promising. According to Derakhshan et al. (2020), commitment to lifelong learning through regularly attending teacher-training courses, doing action research, and participating in teacher professional development conferences are a desideratum for the successful practice of EFL teachers.

### Conclusion and Implications

The current research intended to explore if explicit instruction on teacher classroom justice, presented in a four-session online training course, could enhance Iranian EFL teachers’ justice perceptions and practices. The comparison of the pre- and post-test quantitative results demonstrated that, except for distributive justice, the training that the participants received significantly improved their perceptions of procedural, interactional, and total classroom justice. These findings were in congruence with the content analyses of the qualitative data, which revealed the following themes. First, many teachers confirmed the usefulness of the training that they received on classroom justice. Second, they considered classroom justice more important to them after attending the course. Third, they reported positive changes happening in their knowledge of classroom justice because of the received training. Fourth, they acknowledged learning many justice enactment strategies during the course. Fifth, after trainings, they reported experiencing improvements in the different aspects of their classroom behaviors and practices. Sixth, they showed their willingness to attend more justice trainings in the future.

Based on these results, it can be concluded that training and explicit instruction effectively enhance EFL teachers’ knowledge, perceptions, and practices of classroom justice. These findings empirically support two arguments; first, the teachers’ perceptions and behaviors are quite malleable and open to change (Chory-Assad and Paulsel, 2004; Estaji and Zhaleh, 2021a), and second, the teachers’ perceptions and practices can be improved as a result of effective training (McClellan et al., 2012; Sonnleitner and Kovacs, 2020). The prospect is that these findings enlighten the performance of various stakeholders in the domain of language education.

Accordingly, considering the essentiality of teachers’ just performance in the instructional context (Mameli et al., 2018; Sabbagh, 2021), those authorities in charge of designing curriculum and content for L2 teacher preparation programs should understand the need for running justice-oriented training courses and embodying the knowledge of classroom justice in the instructional materials that they design for their attendees. In the same vein, in line with Coombe and Hiasat’s (2020) recommendation, to increase the teachers’ justice literacy, the educational policymakers are advised to inculcate instructions of justice into graduate and postgraduate programs, encourage the teachers’ engagement with justice-oriented research undertakings, facilitate access to relevant resources and books, and provide the opportunities for participating in courses, forums, workshops, and conferences about classroom justice.

Similarly, L2 teacher educators should update themselves with the most recent findings in the domain of classroom justice research and convey this knowledge to student teachers and in-service teachers who participate in their teacher education programs. In this respect, teacher educators are urged to re-plan their training programs from purely theory-based and universal ones toward more practice-based and contextualized courses, which can meet the actual needs of language teachers, including the need to learn how to act justly in their specific instructional contexts. By doing so, teacher educators can prepare L2 teachers for how to wisely tackle the day-to-day challenges of implementing justice in their classes. Furthermore, taking into account that the teachers’ professional growth is a career-long process, requiring recurrent updating of one’s teaching repertoire (Gutierez and Kim, 2017), it is recommended that L2 teachers voluntarily attend justice-oriented training courses regularly to meet their continuing professional development needs for becoming a just teacher (Derakhshan et al., 2020).

Nevertheless, like any other empirical investigation, this study has some limitations, which can be addressed in future research endeavors. First, due to practicality considerations, the instructions were restricted to 6 h and four sessions. Future studies can prolong such training and see if extending the duration of instructions brings about further improvements. Second, although maximum variation sampling was employed to increase the sample-to-population representativeness of the data, the participants entailed only 77 Iranian EFL teachers. Future studies can replicate this study with larger samples if accessible to increase the transferability of their findings. Third, to collect data, questionnaires were employed, in both close- and open-ended formats. Researchers can utilize other teacher self-evaluation instruments such as a checklist, diary writing, narrative, audio journal, interview, and portfolio to reach more profound insights and compare their results against those obtained in the present study. Fourth, only one group was involved in this research, and within-sample comparisons were made. Future studies can address this issue by dividing their participants into experimental, comparison, and control groups and make within- as well as between-sample comparisons of the data.

Fifth, due to the COVID-19 outbreak, in this study, all the instructional sessions were held online. It is suggested that after the situation returns to normal, the researchers replicate such training in in-person classrooms, where there is more likelihood of face-to-face interactions and collaborations among learners (Derakhshan et al., 2021), and examine if the learning types (online vs. face-to-face) mediate the effect of training on L2 teachers’ justice perceptions and practices. Sixth, the posttest data were collected from the participants a week after the instructions. Future researchers can administer delayed posttest instruments to canvass if the improvements and effects obtained in the study are permanent in the long term. Finally, this study was among the first of its type, which is justifiable when considering that the theoretical and empirical backgrounds of classroom justice are at their nascent stages of development in L2 education. Thus, research evidence has yet to come from other parts of the globe to empirically test the applicability of the social psychology theory of classroom justice, initially introduced in communication education (Chory-Assad and Paulsel, 2004; Resh and Sabbagh, 2016), to the L2 education domain.

## Data Availability Statement

The original contributions presented in the study are included in the article/Supplementary Material, further inquiries can be directed to the corresponding author.

## Author Contributions

All authors listed have made a substantial, direct and intellectual contribution to the work, and approved it for publication.

## Conflict of Interest

The authors declare that the research was conducted in the absence of any commercial or financial relationships that could be construed as a potential conflict of interest.

## Publisher’s Note

All claims expressed in this article are solely those of the authors and do not necessarily represent those of their affiliated organizations, or those of the publisher, the editors and the reviewers. Any product that may be evaluated in this article, or claim that may be made by its manufacturer, is not guaranteed or endorsed by the publisher.

## Supplementary Material

The Supplementary Material for this article can be found online at:

https://www.frontiersin.org/articles/10.3389/fpsyg.2022.821763/full#supplementary-material
