# Supplementary material for: The Enactment of Classroom Justice Through Explicit Instruction: Deciphering the Changes in English as a Foreign Language Teachers’ Perceptions and Practices
Source: Front Psychol. 2022 Feb 18;13:821763. doi: 10.3389/fpsyg.2022.821763 (PMC8894653; doi:10.3389/fpsyg.2022.821763)
Supplement: Supplementary file 1 [file Data_Sheet_1.docx]

**Appendices**

**Appendix A. The Needs Analysis Open-Ended Questionnaire Items**


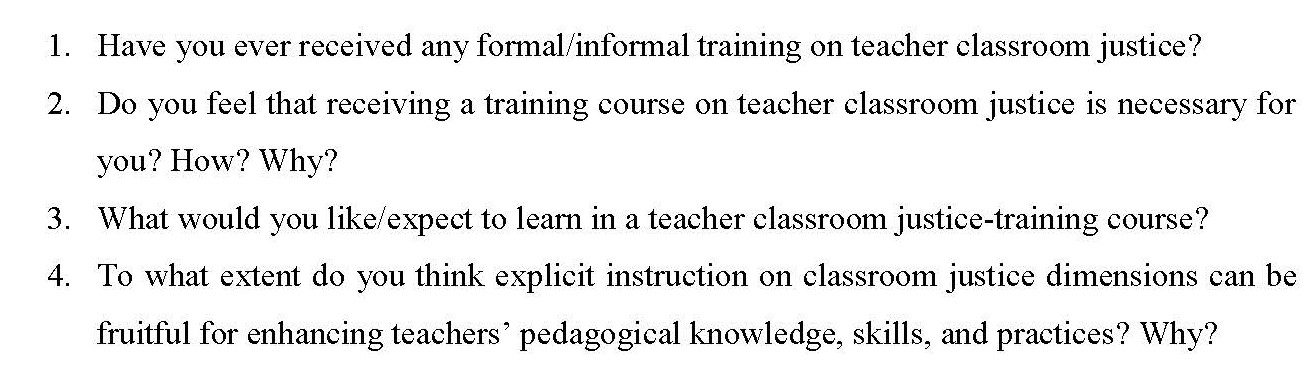


**Appendix B. The Follow-Up Open-Ended Questionnaire Items**

**
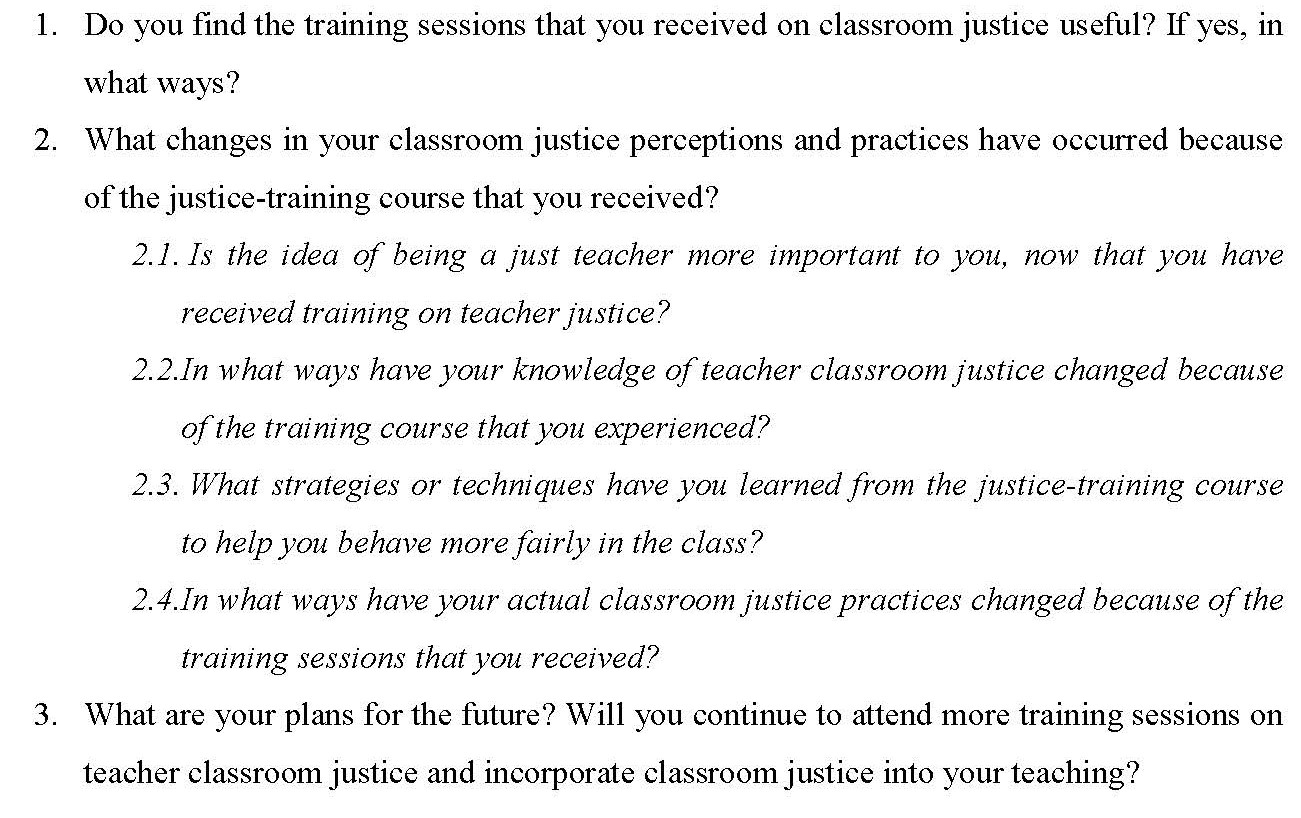
**

**Appendix C. The Syllabus Designed for the Teacher Justice Training Course**

**
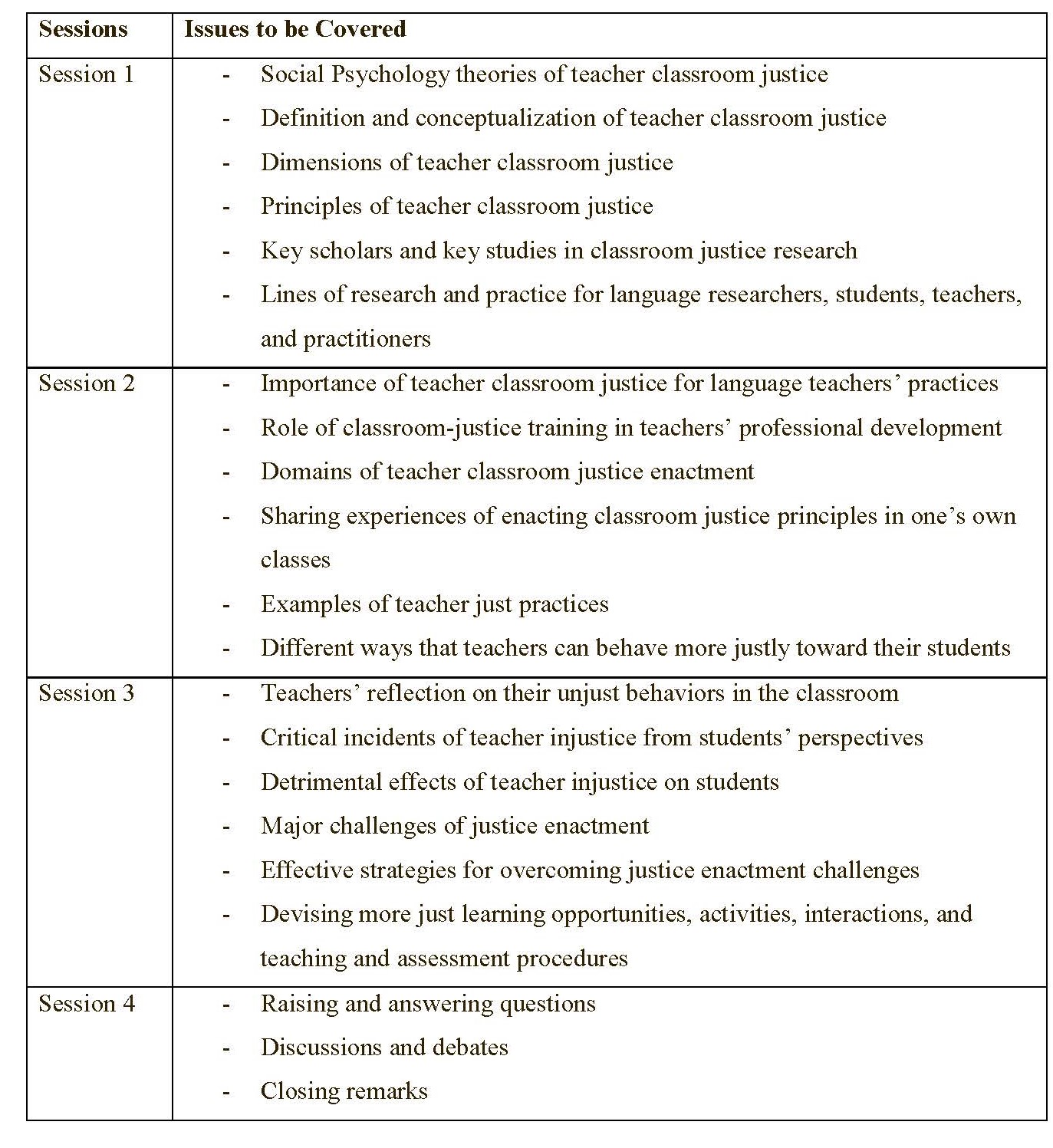
**

**Appendix D. Changes in the Teachers’ Justice Knowledge**


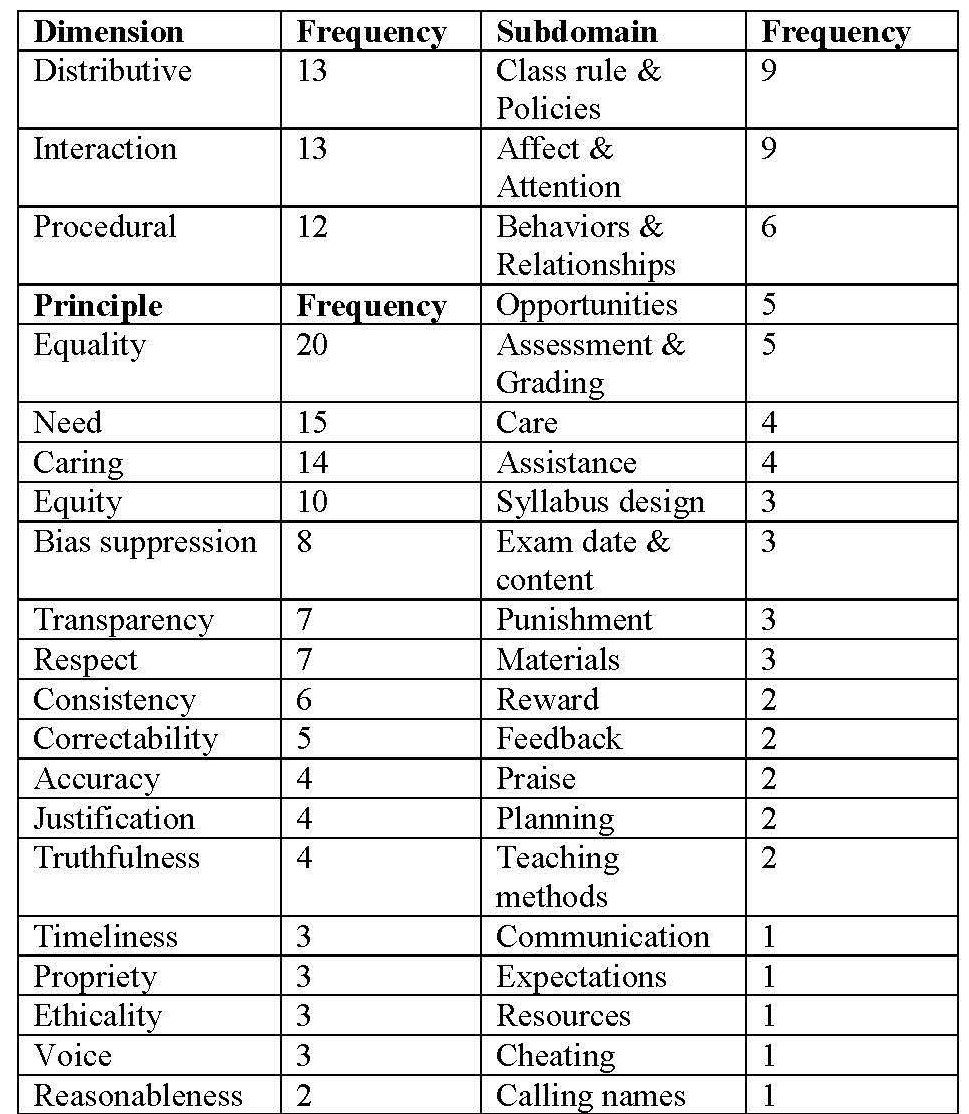


**Appendix E. Justice enactment strategies learned by the teachers**

**
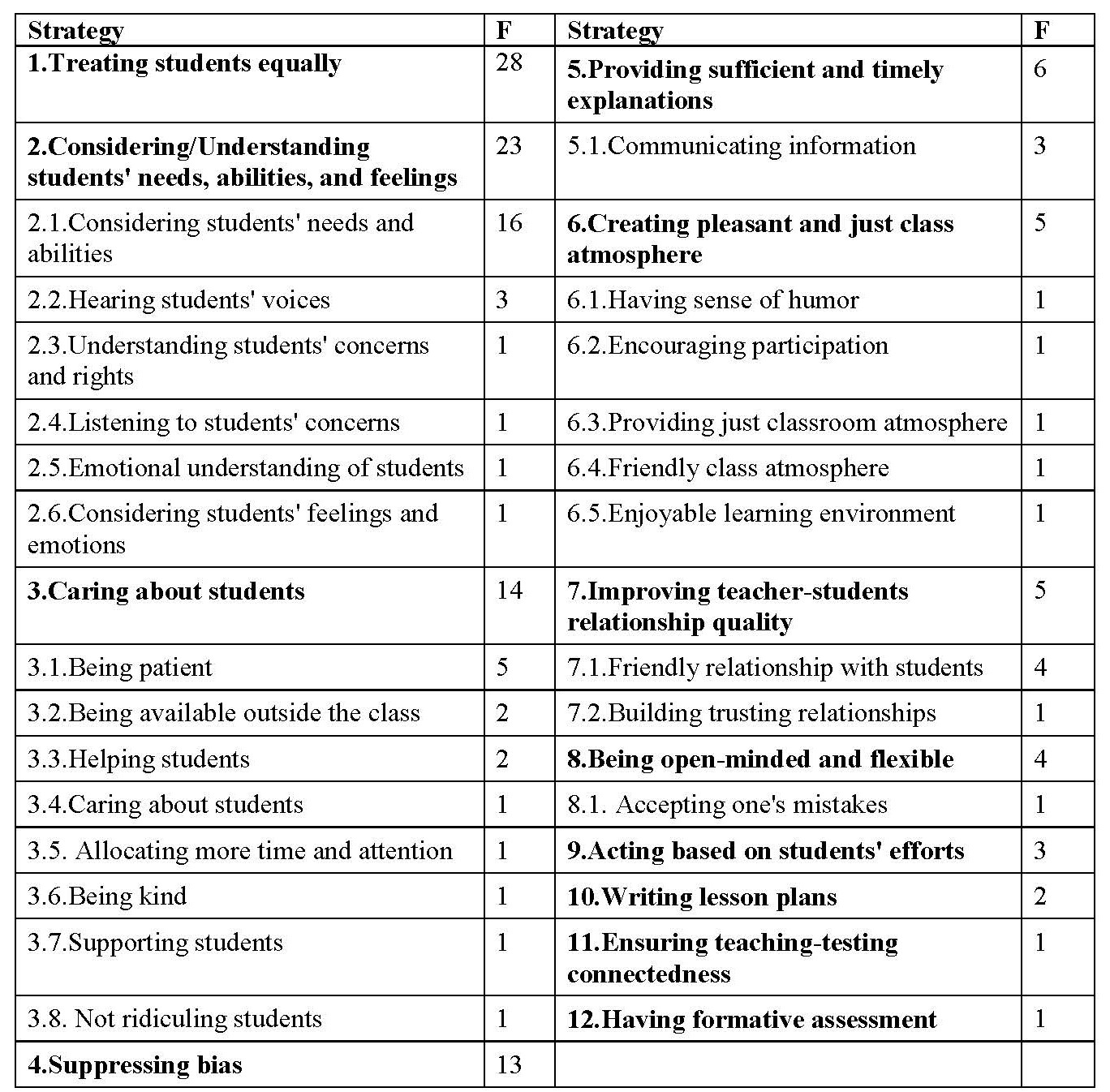
**
